# Fasudil improves survival and promotes skeletal muscle development in a mouse model of spinal muscular atrophy

**DOI:** 10.1186/1741-7015-10-24

**Published:** 2012-03-07

**Authors:** Melissa Bowerman, Lyndsay M Murray, Justin G Boyer, Carrie L Anderson, Rashmi Kothary

**Affiliations:** 1 Ottawa Hospital Research Institute, 501 Smyth Road, Ottawa, ON, Canada K1H 8L6; 2 Department of Cellular and Molecular Medicine, University of Ottawa, 451 Smyth Road, Ottawa, ON, Canada K1H 8M5; 3 Department of Medicine, University of Ottawa, 451 Smyth Road, Ottawa, ON, Canada K1H 8M5

**Keywords:** spinal muscular atrophy, fasudil, survival motor neuron protein, NMJ, muscle

## Abstract

**Background:**

Spinal muscular atrophy (SMA) is the leading genetic cause of infant death. It is caused by mutations/deletions of the survival motor neuron 1 (*SMN1*) gene and is typified by the loss of spinal cord motor neurons, muscular atrophy, and in severe cases, death. The SMN protein is ubiquitously expressed and various cellular- and tissue-specific functions have been investigated to explain the specific motor neuron loss in SMA. We have previously shown that the RhoA/Rho kinase (ROCK) pathway is misregulated in cellular and animal SMA models, and that inhibition of ROCK with the chemical Y-27632 significantly increased the lifespan of a mouse model of SMA. In the present study, we evaluated the therapeutic potential of the clinically approved ROCK inhibitor fasudil.

**Methods:**

Fasudil was administered by oral gavage from post-natal day 3 to 21 at a concentration of 30 mg/kg twice daily. The effects of fasudil on lifespan and SMA pathological hallmarks of the SMA mice were assessed and compared to vehicle-treated mice. For the Kaplan-Meier survival analysis, the log-rank test was used and survival curves were considered significantly different at *P *< 0.05. For the remaining analyses, the Student's two-tail *t *test for paired variables and one-way analysis of variance (ANOVA) were used to test for differences between samples and data were considered significantly different at *P *< 0.05.

**Results:**

Fasudil significantly improves survival of SMA mice. This dramatic phenotypic improvement is not mediated by an up-regulation of Smn protein or via preservation of motor neurons. However, fasudil administration results in a significant increase in muscle fiber and postsynaptic endplate size, and restores normal expression of markers of skeletal muscle development, suggesting that the beneficial effects of fasudil could be muscle-specific.

**Conclusions:**

Our work underscores the importance of muscle as a therapeutic target in SMA and highlights the beneficial potential of ROCK inhibitors as a therapeutic strategy for SMA and for other degenerative diseases characterized by muscular atrophy and postsynaptic immaturity.

## Background

As the leading genetic cause of infant deaths, spinal muscular atrophy (SMA) is a devastating and incurable neuromuscular disorder [1,2]. SMA affects 1 in 6,000 to 10,000 births and results from deletions or mutations in the survival motor neuron 1 (*SMN1*) gene [1-3]. The primary pathological hallmark of SMA is the loss of lower motor neurons from the spinal cord and corresponding muscular atrophy with subsequent paralysis and in most severe cases, death [1,2].

The complete loss of the SMN protein is embryonic lethal [4]. In humans however, a recent duplication event in chromosome 5 has given rise to the centromeric *SMN2 *gene [3]. While both *SMN1 *and *SMN2 *genes differ by only a few nucleotides, a critical C to T substitution lies within position 6 of *SMN2 *exon 7 [5,6]. This silent mutation results in the aberrant splicing of exon 7, giving rise to the biologically unstable SMNΔ7 protein [3,5]. Although the *SMN2 *gene produces predominantly the SMNΔ7 protein, a small amount of full-length SMN is still produced [3]. Thus, the number of *SMN2 *gene copies in SMA patients is a key modifier of disease severity [3,7,8].

One of the major hurdles in SMA is to understand how the loss of a ubiquitously expressed protein leads to the specific loss of spinal cord motor neurons. Work from various research groups has identified distinct roles for SMN in neurodevelopment, neuromaintenance, RNA metabolism, at the neuromuscular junction (NMJ) and in skeletal muscle (reviewed in [9]). As of yet however, none of these various functions of the SMN protein have been recognized as being solely responsible for SMA pathogenesis.

Work from our laboratory has shown that Smn depletion in cellular and mouse models results in altered expression and localization of a number of regulators of actin cytoskeletal dynamics [10-12]. Indeed, analysis of spinal cords from SMA mice revealed a significant increase in active RhoA (RhoA-GTP) [12], a major upstream regulator of the actin cytoskeleton [13]. RhoA-GTP signaling in neuronal cells modulates various cellular functions such as growth, neurite formation, polarization, regeneration, branching, pathfinding, guidance and retraction (reviewed in [14,15]). Our previous work demonstrated that administration of the RhoA/Rho kinase (ROCK) inhibitor Y-27632 [16] leads to a dramatic increase in survival in an intermediate mouse model of SMA [12]. Recently Nölle *et al*. demonstrated that knockdown of Smn in PC12 cells affects the phosphorylation state of downstream effectors of ROCK, supporting the value of the ROCK pathway as a therapeutic target for SMA pathogenesis [17].

In the present work, we have treated SMA mice with fasudil, a ROCK inhibitor approved for US clinical trials. We show that fasudil dramatically improves the lifespan and increases muscle fiber size in *Smn^2B/- ^*SMA mice. Furthermore, we report for the first time that ROCK inhibition restores normal expression of markers of skeletal muscle development in SMA mice. Our study highlights the beneficial effects of ROCK inhibition not only for SMA pathogenesis but also for any degenerative disease that has NMJ and skeletal muscle development defects. Importantly, as fasudil is currently being used in US Food and Drug Administration (FDA)-approved clinical trials for other disorders, re-purposing it is an exciting and feasible therapeutic approach for the treatment of SMA.

## Methods

### Animal models

The *Smn^2B/- ^*mice were established in our laboratory and maintained in our animal facility on a C57BL/6 × CD1 hybrid background. The 2B mutation consists of a substitution of three nucleotides in the exon splicing enhancer of exon 7 [18,19]. The *Smn *knock-out allele was previously described by Schrank *et al*. [20] and *Smn^+/- ^*mice were obtained from The Jackson Laboratory (Bar Harbor, Maine, USA). All animal procedures were performed in accordance with institutional guidelines (Animal Care and Veterinary Services, University of Ottawa).

### Fasudil administration

Fasudil (LC Laboratories; Woburn, Massachusetts, USA) was diluted in water and administered by a modified oral gavage procedure [21] to *Smn^2B/- ^*and *Smn^2B/+ ^*mice from post-natal day (P)3 to P21. The three fasudil dosage regimens were as follows: low dose (30 mg/kg once daily), medium dose (30 mg/kg twice daily), and high dose (30 mg/kg twice daily from P3 to P6; 50 mg/kg twice daily from P7 to P13; 75 mg/kg twice daily from P14 to P21). Vehicle-treated animals received water. Survival and weight were monitored daily.

### Antibodies

The primary antibodies used were as follows: mouse anti-actin (1:800; Fitzgerald; Acton, Massachusetts, USA), mouse anti-Smn (1:5000; BD Transduction Laboratories; Mississauga, Ontario, Canada), rabbit anti-phosphorylated cofilin (1:250; Chemicon; Billerica, Massachusetts, USA), rabbit anti-cofilin (1:500; Millipore; Billerica, Massachusetts, USA), rabbit anti-phosphorylated cofilin 2 (1:500; Cell Signaling; Danvers, Massachusetts, USA), rabbit anti-cofilin 2 (1:500; Millipore), mouse anti-myogenin (1:250; BD Transduction Laboratories), rabbit anti-HB9 (1:50; Abcam; Cambridge, Massachusetts, USA), mouse anti-2H3 (1:250; Developmental Studies Hybridoma Bank; Iowa City, Iowa, USA) and mouse anti-SV2 (1:250; Developmental Studies Hybridoma Bank). The secondary antibodies used were as follows: horseradish peroxidase (HRP)-conjugated goat anti-mouse IgG (1:5000; Bio-Rad; Mississauga, Ontario, Canada), HRP-conjugated goat anti-rabbit IgG (1:5000; Bio-Rad), DyLight goat anti-mouse (1:250; Jackson ImmunoResearch; West Grove, Pennsylvania, USA), goat anti-rabbit biotin-SP-conjugated (1:200; Dako; Burlington, Ontario, Canada)), streptavidin-Cy3-conjugated (1:600; Jackson ImmunoResearch) and Alexa Fluor 680 goat anti-mouse (1:5000; Molecular Probes; Burlington, Ontario, Canada). The α-bungarotoxin (BTX) conjugated to tetramethylrhodamine isothiocyanate was from Molecular Probes (5 μg/mL).

### Immunoblot analysis

Equal amounts of spinal cord and tibialis anterior (TA) muscle tissue extracts were separated by electrophoresis on 10% SDS-polyacrylamide gels and blotted onto nitrocellulose membranes (Amersham; Baie d'Urfe, Quebec, Canada). The membranes were blocked in 5% non-fat milk in TBST (10 mM Tris-HCl pH 8.0, 150 mM NaCl, and 0.1% Tween 20 (Sigma; Oakville, Ontario, Canada)). Membranes were incubated overnight at 4°C with primary antibody, followed by a one-hour incubation with the secondary antibody. All washes were performed with TBST. Signals were visualized using the ECL or the ECL plus detection kit (Amersham). Exposure times were chosen based on the saturation of the highest amounts of protein.

### Hematoxylin and eosin staining

Spinal cord (L1-L2 lumbar regions) and TA muscle sections (5 μm) were deparaffinized in xylene and fixed in 100% ethanol. Following a rinse in water, samples were stained in hematoxylin (Fisher; El Paso, Texas, USA) for three minutes, rinsed in water, dipped 40 times in a solution of 0.02% HCl in 70% ethanol and rinsed in water again. The sections were stained in a 1% eosin solution (BDH; Billerica, Massachusetts, USA) for one minute, dehydrated in ethanol, cleared in xylene and mounted with Permount (Fisher). Images were taken with a Zeiss Axioplan2 microscope, with a 20× objective.

Quantitative assays were performed on three mice for each genotype and five sections per mouse. Motor neurons were identified by their shape and size (> 10 μm in diameter) in the same designated area of the ventral horn region of the spinal cord. Every fifth section was analyzed and the subsequent totals were multiplied by five to give an estimate of total motor neuron number. Only motor neurons with visible nuclei were counted so as to prevent double-counting. For TA quantitative assays, the area of muscle fiber within designated regions of the TA muscle sections was measured using the Zeiss AxioVision software.

### Immunohistochemistry

For immunohistochemistry, spinal cord sections were first deparaffinized in xylene (3 × 10 minutes), fixed in 100% ethanol (2 × 10 minutes), rehydrated in 95% and 75% ethanol (5 seconds each) and placed for 5 minutes in 1 M Tris-HCl pH 7.5. Sections were then placed in boiling sodium citrate antigen retrieval buffer (10 mM sodium citrate, 0.05% Tween 20, pH 6.0) for 20 minutes in the microwave. The sections were rinsed for 10 minutes under running cold tap water and incubated for 2 hours at room temperature (RT) in blocking solution (TBLS (10% NaN_3_), 20% goat serum, 0.3% Triton X-100). This was followed by an overnight incubation at 4°C with the primary antibody. Subsequently, sections were incubated for 1 hour at RT with the biotinylated rabbit antibody followed by a 1 hour incubation at RT with streptavidin-Cy3. All washes were done with PBS. Hoechst (1:1000) was added to the last PBS wash followed by the slides being mounted in fluorescent mounting medium (Dako). Images were taken with a Zeiss confocal microscope, with a 20× objective, equipped with filters suitable for Cy3/Hoechst fluorescence.

### Neuromuscular junction immunohistochemistry

Transversus abdominis (TVA) and TA muscle sections were labeled by immunohistochemistry to allow quantification of neuromuscular innervation as described previously [12,22]. Briefly, TVA muscles were immediately dissected from recently sacrificed mice and fixed in 4% paraformaldehyde (Electron Microscopy Science; Hatfield, Pennsylvania, USA) in PBS for 15 minutes. Post-synaptic acetylcholine receptors were labeled with αBTX for 10 minutes. Muscles were then permeabilized in 2% TritonX for 30 minutes and blocked in 4% bovine serum albumin/1% TritonX in PBS for 30 minutes before incubation overnight in primary antibodies and visualized with DyLight-conjugated secondary antibodies. Whole TA muscles were dissected and fixed in 4% paraformaldehyde. Following the removal of connective tissue, the TA muscles were incubated with αBTX Alexa Fluor 555 conjugate for 20 minutes at RT. Whole TVA muscle and a thin filet of TA muscle were mounted in Dako fluorescent mounting media. Images were taken with a Zeiss confocal microscope equipped with filters suitable for fluorescein isothiocyanate (FITC)/Cy3/fluorescence. Categorization of pre- and postsynaptic morphologies was performed as previously reported [23].

### Pen test

Balance and strength were assessed using the pen test as described [24]. Mice were placed on a suspended pen at different time-points (P12, 14, 17 and 21). The latency to fall from the pen was measured with a plateau of 30 seconds. At each time-point, individual mice were assessed three consecutive times.

### Statistical methods

All statistical analyses were performed using the GraphPad Prism software. For the Kaplan-Meier survival analysis, the log-rank test was used and survival curves were considered significantly different at *P *< 0.05. When appropriate, the Student's two-tail *t *test for paired variables and one-way ANOVA were used to test for differences between samples and data were considered significantly different at *P *< 0.05.

## Results

### Fasudil increases lifespan of Smn^2B/- ^mice

The *Smn^2B ^*allele harbors a sequence change in the exon splicing enhancer of exon 7 of the murine *Smn *gene, leading to the predominant production of the SmnΔ7 protein [18,19]. The *Smn^2B ^*allele in combination with the knockout allele results in an intermediate SMA mouse model (*Smn^2B/-^*) with a median lifespan of 30 days that displays motor neuron loss, neuromuscular defects and immature NMJs [23]. We have previously shown that the ROCK inhibitor Y-27632 dramatically improved the lifespan of *Smn^2B/- ^*mice [12]. Since Y-27632 has not been approved for clinical use, we set out to determine if the ROCK inhibitor, fasudil, which has been approved for US clinical trials [25], would have similar beneficial effects on the *Smn^2B/- ^*mice.

**Figure 1:**
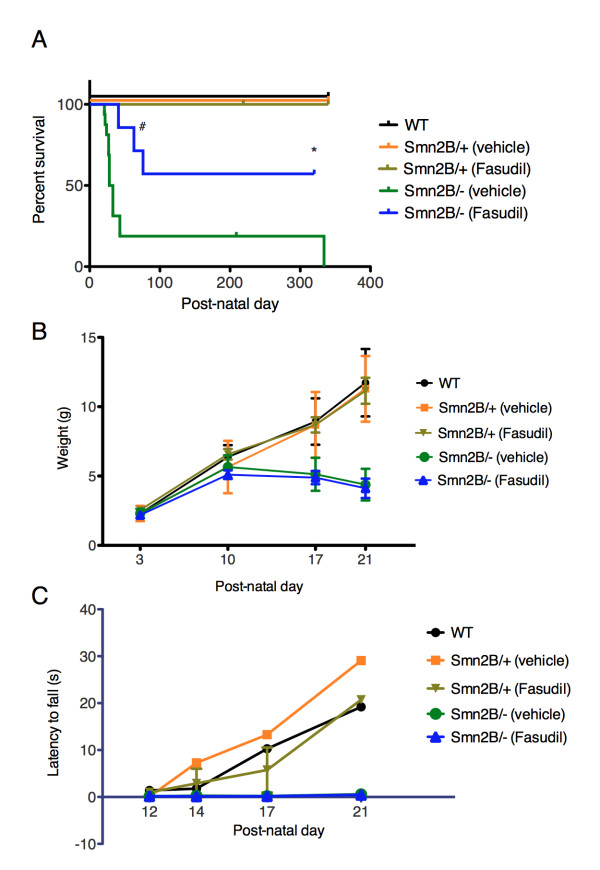
**Fasudil increases lifespan of *Smn^2B/- ^*mice, independent of weight gain and pen test performance**. Fasudil (30 mg/kg twice daily) or vehicle (water) was administered by gavage from post-natal (P) day 3 to P21. The different groups analyzed were: untreated wild type (WT) (n = 10), vehicle-treated *Smn^2B/+ ^*(n = 8), fasudil-treated *Smn^2B/+ ^*(n = 9), vehicle-treated *Smn^2B/- ^*(n = 16) and fasudil-treated *Smn^2B/- ^*(n = 7). (**A**) Fasudil significantly increases lifespan of *Smn^2B/- ^*mice when compared to vehicle-treated *Smn^2B/- ^*mice (*P = 0.0251; # indicates death due to dystocia). Administration of fasudil does not have adverse effects on the lifespan of normal littermates. (**B**) Fasudil does not prevent the arrest in weight gain that occurs in vehicle-treated *Smn^2B/- ^*mice onwards of P10.(**C**) Fasudil does not improve the performance of *Smn^2B/- ^*mice on the pen test when compared to vehicle-treated *Smn^2B/- ^*mice.

Treating the *Smn^2B/- ^*mice by gavage twice daily (30 mg/kg dose) from P3 to P21 led to a significant increase in lifespan when compared to vehicle-treated *Smn^2B/- ^*mice (Figure 1A). Indeed, 57% of fasudil-treated *Smn^2B/- ^*mice survived over 300 days while the median survival of vehicle-treated *Smn^2B/- ^*mice is 30.5 days (Figure 1A). A lower dose of fasudil had no effect while a higher dose was accompanied by a non-negligible toxicity (Additional file 1). Interestingly, both vehicle- and fasudil-treated *Smn^2B/- ^*mice showed a similar arrest in weight gain after P10 (Figure 1B). When assessing strength and balance via the pen test [24], both vehicle- and fasudil-treated *Smn^2B/- ^*mice had short latencies to fall from the pen (Figure 1C). Thus, while fasudil significantly increased the lifespan of the *Smn^2B/- ^*mice, it did not influence the weight loss or the neuromuscular weakness that typifies this SMA mouse model [23]. However, when comparing mice past weaning age, we find that fasudil-treated *Smn^2B/- ^*mice are better groomed, move about more freely in the cage and display a less severe neurological phenotype than vehicle-treated *Smn^2B/- ^*mice (Additional file 2). Additionally, despite their initial compromised body size and neuromuscular function, surviving fasudil-treated *Smn^2B/- ^*female mice are able to reproduce, as exemplified by a female that was euthanized because of dystocia (Figure 1A). The dystocia-linked death, however, highlights the breeding limitations in these aging fasudil-treated mice that still exhibit an SMA neuromuscular phenotype.

### Fasudil activity in the spinal cord does not prevent motor neuron loss in Smn^2B/- ^mice

**Figure 2:**
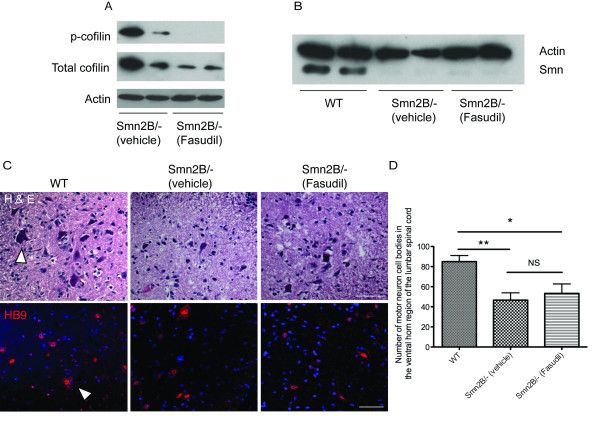
**Fasudil activity in the spinal cord is Smn-independent and does not prevent motor neuron loss in the ventral horn region**. (**A **and **B**) Spinal cords were obtained from post-natal (P) day 21 untreated wild type (WT), vehicle-treated *Smn^2B/- ^*and fasudil-treated *Smn^2B/- ^*mice. (A) Immunoblot analysis shows that spinal cords treated with the rho-kinase (ROCK) inhibitor fasudil have decreased levels of p-cofilin, a known substrate of ROCK. (B) Immunoblot analysis shows that fasudil does not increase Smn protein levels in the spinal cords of *Smn^2B/- ^*mice. (**C **and **D**) Spinal cord sections were analyzed from P21 untreated wild type (WT) (n = 3), vehicle-treated *Smn^2B/- ^*(n = 3) and fasudil-treated *Smn^2B/- ^*(n = 3) mice. (C) Representative images of hematoxylin and esoin (H&E)- and HB9-stained spinal cord sections. Arrowhead depicts a typical large motor neuron. Scale bar = 50 μm. (D) Quantification of motor neurons within the ventral horn region of the spinal cord shows that fasudil does not prevent the motor neuron loss that occurs in vehicle-treated *Smn^2B/- ^*mice (*P < 0.05; **P < 0.01; NS = not significant; data are mean +/- s.d.).

The main goal of using fasudil as a therapeutic strategy is to compensate for the increased levels of RhoA-GTP in the spinal cords of the *Smn^2B/- ^*mice [12]. In order to investigate the mechanisms by which fasudil exerts its beneficial effects, we investigated its activity and impact on motor neuron loss in the spinal cord. Spinal cord extracts from P21 fasudil-treated *Smn^2B/- ^*mice showed a reduction in phosphorylated cofilin, a downstream effector of ROCK [26,27], when compared to vehicle-treated SMA mice (Figure 2A), demonstrating that oral administration of fasudil efficiently delivers the drug to the CNS and leads to an efficient inhibition of ROCK activity.

To investigate if the beneficial effects of fasudil administration are mediated through an increase in Smn expression, we compared Smn protein levels in P21 spinal cords of wild type, vehicle-treated and fasudil-treated *Smn^2B/- ^*mice. This comparison shows that fasudil does not lead to a significant upregulation of Smn expression (Figure 2B) and further suggests that fasudil acts via an Smn-independent pathway to improve the survival of *Smn^2B/- ^*mice.

As a major hallmark of SMA is loss of lower motor neuron cell bodies from the spinal cord, we assessed the effect of fasudil on the motor neuron loss previously characterized in the *Smn^2B/- ^*mouse model [11,23]. Quantification of the number of motor neuron cell bodies in the ventral horn region of L1-L2 lumbar spinal cord sections revealed similar significant reductions in both vehicle- and fasudil-treated *Smn^2B/- ^*mice compared to wild type controls (Figure 2C, D). This implies that the beneficial effects observed following fasudil administration are not mediated via a preservation of motor neuron cell bodies. It is therefore possible that fasudil acts on other SMA-afflicted tissues and/or compartments that subsequently influence the functionality of the surviving motor neurons.

### Fasudil increases skeletal muscle fiber size

**Figure 3:**
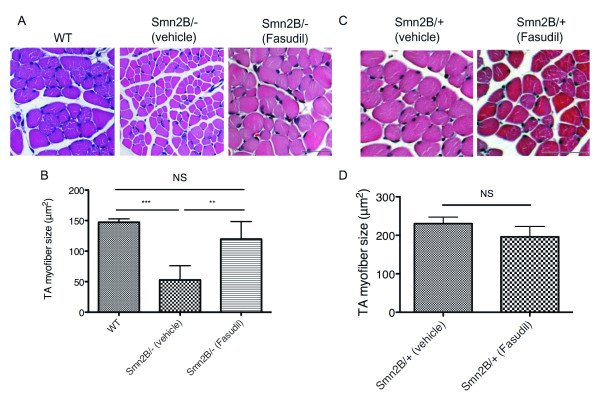
**Fasudil increases tibialis anterior (TA) myofiber size**. TA muscles were isolated from post-natal (P) day 21 untreated wild type (WT) (n = 3), vehicle-treated *Smn^2B/+ ^*(n = 3), fasudil-treated *Smn^2B/+ ^*(n = 3), vehicle-treated *Smn^2B/- ^*(n = 6) and fasudil-treated *Smn^2B/- ^*(n = 6) mice. (**A**) Representative images of cross-sections of WT, or vehicle- and fasudil-treated *Smn^2B/- ^*TA muscles stained with hematoxylin and eosin. Scale bar = 50 μm. (**B**) Quantification shows that fasudil-treated *Smn^2B/- ^*TA muscles display significantly larger myofibers than vehicle-treated *Smn^2B/- ^*mice. (**P < 0.01; ***P < 0.001; NS = not significant; data are mean +/- s.d.). (**C**) Representative images of cross-sections of vehicle- and fasudil-treated *Smn^2B/+ ^*TA muscles stained with hematoxylin and eosin. Scale bar = 50 μm. (**D**) Quantification shows that fasudil does not significantly increase the myofiber size of *Smn^2B/+ ^*normal mice (NS = not significant; data are mean +/- s.d.).

In addition to motor neuron degeneration, SMA is also typified by muscular atrophy [1,2]. In recent years, several intrinsic pathologies and defective molecular pathways have been reported in SMA muscle ([28-30] and JGB, unpublished data). Furthermore, we have previously demonstrated that the ROCK inhibitor Y-27632 leads to an increase in the TA myofiber size of *Smn^2B/- ^*mice [12]. We thus investigated the effect of fasudil on skeletal muscle and show that TA muscles from fasudil-treated P21 *Smn^2B/- ^*mice display significantly larger myofibers than vehicle-treated *Smn^2B/- ^*mice (Figure 3A, B). Indeed, both wild type and fasudil-treated *Smn^2B/- ^*mice show similar myofiber sizes (Figure 3A, B). To determine whether this increase in muscle fiber size was SMA-dependent, we also compared TA muscles of vehicle- and fasudil-treated *Smn^2B/+ ^*phenotypically normal littermates. This revealed no significant difference in myofiber size (Figure 3C, D), thus suggesting that fasudil acts on muscle-specific molecular pathways that are distinctly perturbed in the SMA mice.

### Fasudil-treated muscles display restored myogenin expression

**Figure 4:**
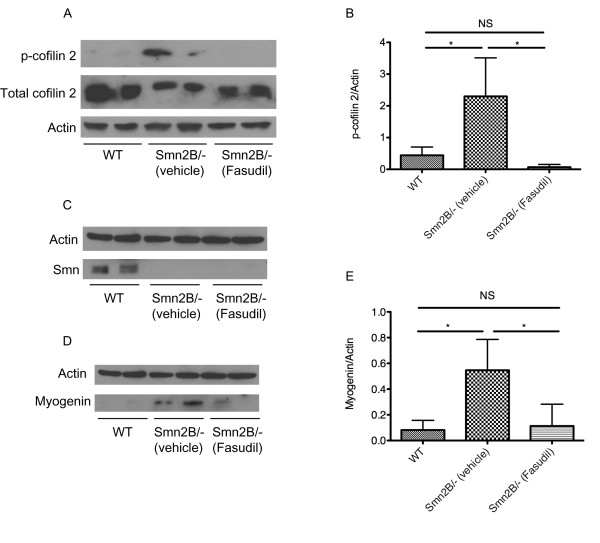
**Fasudil administration inhibits ROCK in skeletal muscle and restores normal myogenin expression levels**. Tibialis anterior (TA) muscles were isolated from post-natal (P) day 21 untreated wild type (WT) (n = 4), vehicle-treated *Smn^2B/- ^*(n = 4) and fasudil-treated *Smn^2B/- ^*(n = 4) mice. (**A**) Immunoblot analysis of p-cofilin 2, a muscle-specific downstream substrate of ROCK, shows that its levels are increased in the TA muscles of *Smn^2B/- ^*mice compared with wild type. Furthermore, this experiment shows that fasudil inhibits ROCK in the TA muscle of *Smn^2B/- ^*mice, and reduces the p-cofilin 2 levels to wild type levels. (**B**) Quantification shows that wild type TA muscles have significantly less p-cofilin 2 than vehicle-treated *Smn^2B/- ^*TA muscles, and that fasudil is active in skeletal muscle and restores p-cofilin 2 to normal levels. (*P < 0.05; NS = not significant; data are mean +/- s.d.). (**C**) Immunoblot analysis shows that fasudil does not increase Smn protein levels in the TA muscles of *Smn^2B/- ^*mice. (**D**) Immunoblot analysis shows that fasudil results in a decrease in myogenin protein levels in fasudil-treated *Smn^2B/- ^*TA muscles when compared to vehicle-treated *Smn^2B/- ^*mice. (**E**) Wild type muscle has significantly less myogenin than vehicle-treated *Smn^2B/- ^*TA muscles. Fasudil administration to *Smn^2B/- ^*mice restores myogenin to normal levels (*P < 0.05; NS = not significant; data are mean +/- s.d.).

To assess if fasudil was active in skeletal muscle, we examined factors downstream of ROCK signaling. Cofilin 2 is a skeletal muscle-specific actin-regulating protein and downstream effector of activated ROCK [31,32]. We thus determined the impact of administrating fasudil by gavage on skeletal muscle by evaluating p-cofilin 2 levels in vehicle- and fasudil-treated TA muscles from P21 mice. Interestingly, the TA muscles from *Smn^2B/- ^*mice have significantly higher levels of p-cofilin 2 protein than wild type muscles (Figure 4A, B), suggesting that the RhoA/ROCK pathway is also misregulated in skeletal muscle. Fasudil decreases p-cofilin 2 levels in *Smn^2B/- ^*muscle to wild type levels, indicating that it is active in the TA muscle and restores the normal ROCK/p-cofilin 2 levels (Figure 4A, B). We also show that fasudil does not upregulate Smn expression in the TA muscles of *Smn^2B/- ^*mice (Figure 4C). Thus, consistent with the spinal cord analysis (Figure 2B), it appears that the beneficial effects of fasudil in skeletal muscle are most likely Smn-independent.

Recent work from our laboratory suggests that hindlimb muscles from P21 *Smn^2B/- ^*mice display defects in muscle development, as evidenced by the misregulation of myogenin (JGB, unpublished data), a transcription factor that plays a well-characterized role in myogenesis [33]. We thus investigated whether fasudil had any impact on myogenin levels. Analysis of TA muscles from P21 mice confirms the increased levels of myogenin in skeletal muscle of *Smn^2B/- ^*mice compared to wild type controls (Figure 4D, E). Importantly, fasudil administration leads to a significant decrease in myogenin levels in *Smn^2B/- ^*mice (Figure 4D, E). In fact, myogenin levels in fasudil-treated TA muscles are restored to wild type levels. Thus, fasudil returns the expression level of myogenin to normal, suggesting that fasudil may increase muscle size by restoring the typical development of skeletal muscle.

### Fasudil does not ameliorate the pre-synaptic phenotype of NMJs from Smn^2B/- ^mice

**Figure 5:**
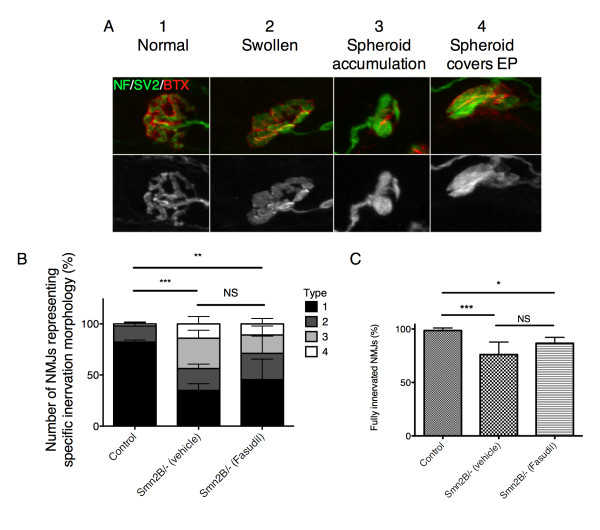
**Fasudil does not improve pre-synaptic neuromuscular junction (NMJ) phenotype of *Smn^2B/- ^*mice**. Pre-synaptic morphology was analyzed in the transversus abdominis (TVA) muscle of post-natal (P) day 21 untreated control littermates (n = 3), vehicle-treated *Smn^2B/- ^*(n = 6) and fasudil-treated *Smn^2B/- ^*(n = 4) mice. (**A**) Representative images of NMJs depicting the pre-synaptic morphology categories: normal (type 1), swollen (type 2), spheroid accumulation (type 3) and spheroids covers the endplate (EP) (type 4). (Neurofilament (NF) and synaptic vesicle protein 2 (SV2): green; EP: red (BTX)). (**B**) Quantification of the pre-synaptic morphology shows that control littermates have significantly more 'normal' (type 1) NMJs than both vehicle- and fasudil-treated *Smn^2B/- ^*mice (**P < 0.01; ***P < 0.001; NS = not significant; data are mean +/- s.d.). C) Quantification of the fully innervated NMJs shows that both vehicle- and fasudil-treated *Smn^2B/- ^*muscles display significantly fewer fully innervated NMJs than control littermates, with no significant difference between vehicle or Ffsudil treated *Smn^2B/- ^*mice (*P < 0.05; ***P < 0.001; NS = not significant; data are mean +/- s.d.). BTX, α-bungarotoxin.

We have previously identified pre-synaptic pathology at the NMJ in the TVA of the *Smn^2B/- ^*mice, as evidenced by a loss of pre-synaptic inputs and accumulation of neurofilaments [23]. To determine if the reduction in muscle pathology observed following fasudil administration could be secondary to reduced pathology at the NMJ, an in-depth analysis was performed. This analysis was done on the TVA muscle of P21 late-symptomatic mice, which has previously been shown to display marked NMJ loss and pre-synaptic abnormalities [23]. The degree of pre-synaptic swelling, identified by neurofilament (NF) and synaptic vesicle 2 (SV2) staining, was classified into four categories based on morphology (type 1: normal, no pre-synaptic swelling observed; type 2: swollen, pre-synaptic terminal arborization is thickened compared to type 1; type 3: spheroid accumulations, spherical swellings accumulate over the NMJ; type 4: spheroid covers endplate (EP), spherical accumulations obscure the entire EP) (Figure 5A). While more than 80% of wild type terminals displayed a 'normal' pre-synaptic morphology, in both vehicle- and fasudil-treated *Smn^2B/- ^*mice we observed a high level of pre-synaptic swelling, with more than 50% of EPs displaying morphologies classified as types 2 to 4 (Figure 5B). Quantification of the number of fully innervated NMJs shows a significant decrease in both vehicle- and fasudil-treated *Smn^2B/- ^*mice when compared to wild type, with no significant difference observed between vehicle- and fasudil-treated SMA mice (Figure 5C). Thus, it appears, at least in P21 animals, that fasudil does not ameliorate the pre-synaptic phenotype observed in *Smn^2B/- ^*mice. This suggests that the improvement in muscle pathology we observe after fasudil administration is unlikely to be mediated through the NMJ, and that it likely is having a direct effect on the muscle.

### Fasudil increases endplate area of Smn^2B/- ^NMJs

**Figure 6:**
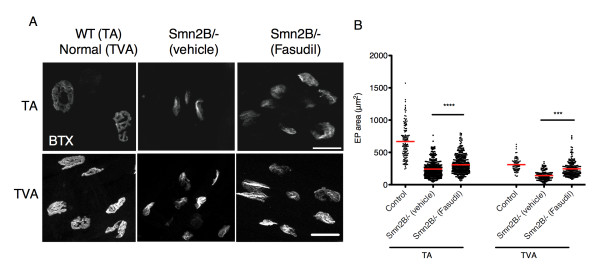
**Fasudil increases endplate (EP) area in the tibialis anterior (TA) and transversus abdominis (TVA) muscles**. Muscles were isolated from post-natal (P) day 21 untreated control littermates (n = 3), vehicle-treated *Smn^2B/- ^*(n = 4) and fasudil-treated *Smn^2B/- ^*mice (n = 3). (**A**) Representative images of TA and TVA EPs stained with α-bungarotoxin (BTX). Scale bars = 25 μm (TA) and 30 μm (TVA). (**B**) Quantification of EP area shows that fasudil-treated *Smn^2B/- ^*TA and TVA muscles display significantly larger EPs when compared to vehicle-treated *Smn^2B/- ^*muscles. (***P < 0.001; ****P < 0.0001; data are mean +/- s.d.).

We have previously shown that Y-27632 administration significantly increases the EP area of NMJs within the TA skeletal muscle [12]. We thus assessed the effect of fasudil on EP size in both the TA and the TVA of P21 mice. Fasudil-treated *Smn^2B/- ^*mice had significantly larger TA and TVA EP areas than vehicle-treated *Smn^2B/- ^*mice (Figure 6). Interestingly, this increase in EP area was weight-independent, since both vehicle- and fasudil-treated *Smn^2B/- ^*mice display similar weight curves (Figure 1B). Our results suggest that while fasudil does not ameliorate the pathology evident at the pre-synaptic compartment of P21 *Smn^2B/- ^*NMJs (Figure 5), there is a dramatic increase in the size of the post-synaptic compartment of NMJs within both muscles investigated (Figure 6). Together, these results are consistent with the beneficial effects of fasudil being primarily mediated by the muscle and not the motor neuron.

### Increased NMJ maturation is observed in aging fasudil-treated Smn^2B/- ^mice

**Figure 7:**
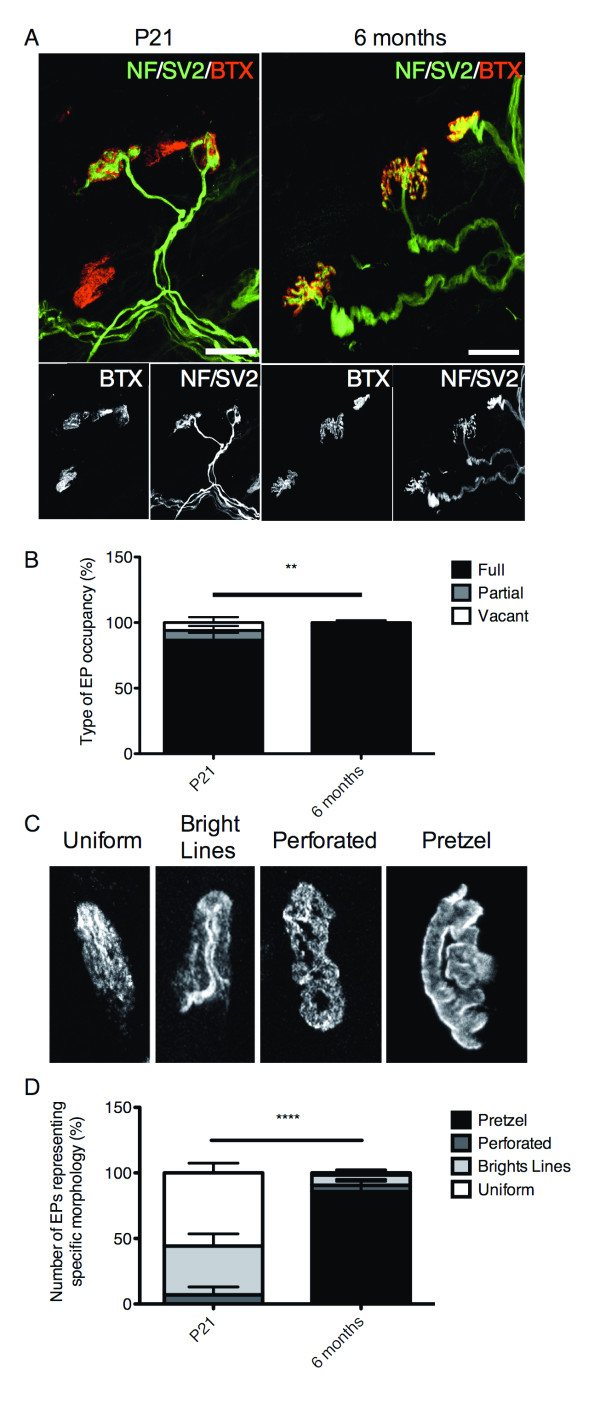
**Aging fasudil-treated *Smn^2B/- ^*mice display mature neuromuscular junctions (NMJs)**. Transversus abdominis (TVA) muscles were isolated from post-natal (P) day 21 (n = 6) and 6-month -old (n = 4) fasudil-treated *Smn^2B/- ^*mice. (**A**) Representative images of TVA muscles from P21 and 6-month-old fasudil-treated *Smn^2B/- ^*mice. (Neurofilament (NF) and synaptic vesicle protein 2 (SV2): green; EP: red (BTX)). Scale bar = 30 μm. B) Types of EP occupation were categorized and quantified as fully occupied (where the pre-synaptic terminal completely covers the EP), partial (where the pre-synaptic terminal partially covers the EP), or vacant (where no pre-synaptic terminal is present at an EP). Surviving 6-month-old fasudil-treated *Smn^2B/- ^*mice display significantly more fully occupied EPs than P21 fasudil-treated *Smn^2B/- ^*mice (**P = 0.0096; data are mean +/- s.d.). **(C**) Representative images of EP morphology categorization from mature to immature: pretzel, perforated, bright lines and uniform. EPs are visualized with BTX. (**D**) Quantification shows that 6-month-old fasudil-treated *Smn^2B/- ^*mice display significantly more mature pretzel-shaped EPs than P21 fasudil-treated *Smn^2B/- ^*mice (****P < 0.0001); data are mean +/- s.d.). BTX, α-bungarotoxin; EP, endplate.

Although we saw no improvement in pre-synaptic NMJ pathology in P21 *Smn^2B/- ^*mice, we wanted to evaluate the effect of fasudil over time on NMJ pathology. NMJs from the TVA muscle of P21 Fasudil-treated *Smn^2B/- ^*mice were compared to those of six month old fasudil-treated *Smn^2B/- ^*mice. Interestingly, we observed a marked decrease in pre-synaptic pathology in six month old mice compared to P21 mice, as evidenced by an increase in the percentage of fully occupied EPs (Figure 7A, B). This was accompanied by a dramatic increase in EP maturation (Figure 7C, D). We, therefore, suggest that although there was no initial improvement in the morphological aspects on NMJ pathology, given sufficient time, fasudil administration allows for the improved maturation of NMJs in *Smn^2B/- ^*mice.

## Discussion

Previous work has implicated the RhoA/ROCK pathway in SMA pathogenesis [10,12,17]. In the present study, we demonstrate that targeting the ROCK pathway with the inhibitor fasudil significantly increases the lifespan of the *Smn^2B/- ^*SMA mice. The increased survival is independent of Smn expression, weight gain, pen test performance and pre-synaptic NMJ phenotype. We find, however, that fasudil benefits post-synaptic pathology and muscle development. Importantly, the results obtained from other fasudil clinical trials are proof-of-principle of its feasibility and availability as a therapeutic approach for the treatment of SMA. Future SMA clinical endeavors should therefore consider assessing the beneficial potential of ROCK inhibitors.

Smn protein levels remained significantly low in both fasudil-treated spinal cord and muscle samples of SMA mice. These findings are important when considering therapeutic avenues for SMA. There are presently many strategies being developed to increase the expression of SMN, such as gene therapy, modulation of transcription and splicing of *SMN2*, and the use of various histone deacetylase (HDAC) inhibitors (reviewed in [34-36]). Although these therapeutic approaches show promising results, they remain in pre-clinical stages and may not be as efficient if administered to mid- to late-symptomatic patients [37]. It is therefore crucial to understand the pathological molecular pathways that are affected upon SMN loss and how these can be modulated to attenuate their degenerative effects. Along with other research groups, we have shown that the RhoA/ROCK pathway is indeed perturbed in SMA cellular and animal models and that its targeting leads to a significant beneficial outcome [10,12,17].

We had previously identified the upregulation of RhoA-GTP in the spinal cords of *Smn^2B/- ^*mice [12]. The misregulated RhoA/ROCK pathway in the spinal cord was, therefore, the primary target of our Fasudil therapeutic strategy [12]. Interestingly, we have observed that fasudil does not prevent the motor neuron loss that occurs in the *Smn^2B/- ^*mice. In fact, the most apparent effects of fasudil appear to be the restoration of normal skeletal muscle growth and development, as well as increased post-synaptic EP area. A number of recent reports suggest that the SMN protein may have a muscle-intrinsic role that influences SMA pathology ([28-30] and JGB, unpublished data). Active RhoA has previously been shown to positively regulate the expression of myogenin [38,39]. Furthermore, work performed in avian and murine myoblasts shows that inhibition of ROCK promotes exit from the cell cycle and subsequent terminal differentiation [40]. Indeed, myoblasts treated with the ROCK inhibitor Y-27632 display increased differentiation, cell fusion and myotube formation [40]. Fasudil's inhibition of the RhoA/ROCK pathway most likely restores the normal skeletal muscle developmental program of *Smn^2B/- ^*mice via modulation of myoblast differentiation and fusion, as well as myogenin expression. The fasudil-dependent increase in myofiber size could lead to the subsequent increase in EP size. Indeed, a positive correlation has previously been established between myofiber size and motor EP size [41]. Furthermore, various reports suggest that post-synaptic differentiation and formation is initially muscle-dependent and motor axon-independent [42,43]. Our study, therefore, highlights two important points. Firstly, therapeutic strategies that improve skeletal muscle and EP growth should be considered when developing therapies for SMA. Secondly, ROCK inhibition may have positive outcomes in other pre-clinical disease models characterized by muscle atrophy and NMJ pathology.

Intriguingly, the dramatic increase in skeletal muscle myofiber size of fasudil-treated *Smn^2B/- ^*mice is not accompanied by changes in weight or strength, when compared to vehicle-treated *Smn^2B/- ^*mice. Previous studies have reported this phenomenon, providing a variety of potential explanations. In cases of sarcoplasmic hypertrophy, the non-contractile myofiber components expand while muscular strength remains unchanged [44]. Further, the characterization of a post-natal myogenin knockout mouse model revealed normal skeletal muscle size albeit with a 30% weight loss compared to control littermates [45]. The authors suggest that this phenotype is caused by a slower growth rate and perturbed energy homeostasis [45]. Finally, Rehfeldt *et al*. showed that mice homozygous for the *Compact myostatin *mutation (*C/C*) display muscular hyperplasia and increased muscle weight but with a reduction in overall body weight [46]. The authors also identify a reduction in the number of capillaries per muscle in the *C/C *mice, subsequently impacting oxidative metabolism [46]. Interestingly, recent work in the severe SMA mouse model demonstrated a significant decrease in the capillary bed density within skeletal muscle [47]. Thus, the findings mentioned above highlight the fact that an increase in muscle size and or weight does not necessarily positively correlate with an increase in body weight. Regardless, the restoration of myofiber growth and skeletal muscle development by fasudil, in the absence of weight gain, appears to be sufficient to provide therapeutic benefits to the *Smn^2B/- ^*mice.

In recent years, it has been postulated that SMA may be a die-back neuropathy, where the motor axons initially reach the EP but subsequently retract as disease progresses [48-50]. This hypothesis suggests that synapses are selectively vulnerable in SMA, with synapse loss preceding cell body degeneration. In addition, it has been suggested that neurons undergo compartmental degeneration, where the soma, axons and synapses of neurons possess specific and compartmentalized mechanisms of degeneration [51-53]. It therefore follows that therapeutics which target distal compartments of the cell, such as the synapse or axon, can be protective to the cell body. In our study, we show that while fasudil administration has little impact upon the initial loss of motor neurons, it dramatically increases myofiber and EP size in SMA mice. We therefore suggest that this improvement in post-synaptic parameters stabilizes the synaptic connections and subsequently protects the remaining motor neurons. Consistent with this observation, the surviving synapses constitute NMJs that will eventually develop and mature normally. Given the tight correlation between EP maturation and neuromuscular activity (reviewed in [54]), fasudil may indirectly improve NMJ transmission, subsequently ameliorating motor EP maturation. Alternatively, considering the crucial role of the actin cytoskeleton in the redistribution of acetylcholine receptors (AChRs) during post-synaptic remodeling [55,56], fasudil's modulation of actin dynamics could directly restore normal AChR clustering. Clearly, the understanding and identification of fasudil's influence on NMJ maturation in SMA mice requires further investigation. Nevertheless, our work highlights the applicability of the compartmental degeneration hypothesis to SMA pathogenesis and the potential of therapies aimed at preventing synaptic degeneration.

ROCK has evolved as an important therapeutic target in various models of cardiovascular disease, spinal cord injury and glaucoma (reviewed in [57-59]). Furthermore, the ROCK inhibitor fasudil, which has been approved in US clinical trials, has shown beneficial effects in patients with vasospastic angina [60], stable effort angina [61], general heart failure [62] and pulmonary hypertension [63]. It has now become evident that the pathogenic misregulation of the RhoA/ROCK pathway in various Smn-depleted cellular and animal models can also be modulated by the ROCK inhibitors Y-27632 and fasudil, leading to significant positive outcomes [10,12,17].

## Conclusions

The administration of fasudil to SMA mice significantly increases their lifespan without an obvious increase in Smn expression or preservation of spinal cord motor neurons. In fact, fasudil improves post-synaptic and skeletal muscle development. Our work underscores the importance of muscle as a therapeutic target in SMA and highlights the beneficial potential of ROCK inhibitors as a therapeutic strategy for SMA and for other degenerative diseases characterized by muscular atrophy and postsynaptic immaturity.

## Abbreviations

α-BTX: α-bungarotoxin; AchR: acetylcholine receptor; CNS: central nervous system; EP: endplate; FDA: Food and Drug Administration; HDAC: histone deacetylase; NF: neurofilament; NMJ: neuromuscular junction; ROCK: Rho-kinase; SMA: spinal muscular atrophy; SMN: survival motor neuron; SV2: synaptic vesicle 2; TA: tibialis anterior; TVA: transversus abdominis.

## Competing interests

The authors declare that they have no competing interests.

## Authors' contributions

MB, LMM and RK conceived and designed the experiments. MB, LMM and CLA performed the experiments. MB and LMM analyzed the data. JGB performed the initial characterization of misregulated myogenin expression in SMA skeletal muscle. MB and LMM wrote the paper. All authors read and approved the final manuscript.

## Pre-publication history

The pre-publication history for this paper can be accessed here:

http://www.biomedcentral.com/1741-7015/10/24/prepub

## Supplementary Material

Additional file 1 **Effect of low and high doses of fasudil**. A low dose of fasudil (30 mg/kg once daily), a high dose of fasudil (30 mg/kg twice daily from P3-P6; 50 mg/kg twice daily from P7-P13; 75 mg/kg twice daily from P14-P21), or vehicle (water) was administered by gavage. The different groups analyzed were: untreated wild type (WT) (n = 10), fasudil (high dose)-treated *Smn^2B/+ ^*(n = 16), vehicle-treated *Smn^2B/- ^*(n = 16), fasudil (low dose)-treated *Smn^2B/- ^*(n = 6) and fasudil (high dose)-treated *Smn^2B/- ^*(n = 10) mice. A) Survival curves show that the low fasudil dosage regimen had no effect while the high fasudil dosage regimen significantly increases the lifespan of *Smn^2B/- ^*mice when compared to vehicle-treated *Smn^2B/- ^*mice (*P = 0.03; # indicates death due to malocclusion of the teeth). B) Survival curve shows that the high fasudil dosage regimen has non-negligible toxic effects on both *Smn^2B/- ^*mice and the normal *Smn^2B/+ ^*littermates. Click here for file

Additional file 2 ***Smn^2B/- ^*mice treated with fasudil show improved gait and movement**. A movie of aging fasudil- (left mouse) and vehicle-treated (right mouse) *Smn^2B/- ^*mice (two months of age). The fasudil-treated mouse displays the agility and ability to freely walk and move around the cage while still displaying a minor neurological phenotype. The vehicle-treated *Smn^2B/- ^*mouse displays a severe neurological phenotype and reduced motor functions. Click here for file
