# Supplementary figures and images for: Fasudil improves survival and promotes skeletal muscle development in a mouse model of spinal muscular atrophy
Source: BMC Med. 2012 Mar 7;10:24. doi: 10.1186/1741-7015-10-24 (PMC3310724; doi:10.1186/1741-7015-10-24)

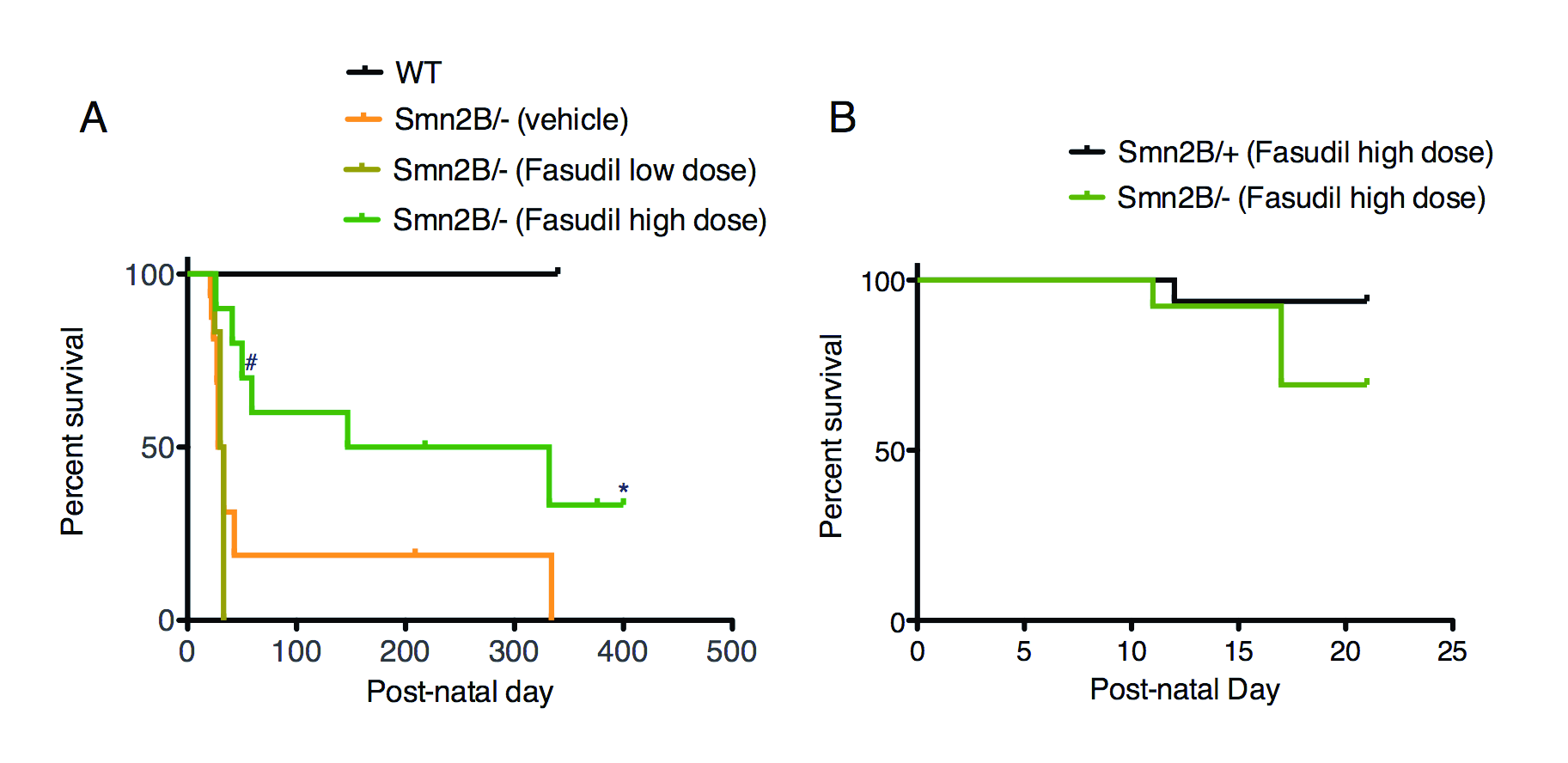

Supplement: Additional file 1 — Effect of low and high doses of fasudil. A low dose of fasudil (30 mg/kg once daily), a high dose of fasudil (30 mg/kg twice daily from P3-P6; 50 mg/kg twice daily from P7-P13; 75 mg/kg twice daily from P14-P21), or vehicle (water) was administered by gavage. The different groups analyzed were: untreated wild type (WT) (n = 10), fasudil (high dose)-treated Smn2B/+ (n = 16), vehicle-treated Smn2B/- (n = 16), fasudil (low dose)-treated Smn2B/- (n = 6) and fasudil (high dose)-treated Smn2B/- (n = 10) mice. A) Survival curves show that the low fasudil dosage regimen had no effect while the high fasudil dosage regimen significantly increases the lifespan of Smn2B/- mice when compared to vehicle-treated Smn2B/- mice (*P = 0.03; # indicates death due to malocclusion of the teeth). B) Survival curve shows that the high fasudil dosage regimen has non-negligible toxic effects on both Smn2B/- mice and the normal Smn2B/+ littermates. [file 1741-7015-10-24-S1.TIFF]
